# 3D Printed Microfluidic Chromatographic Column for Fast Downstream Processing Development

**DOI:** 10.1002/biot.70095

**Published:** 2025-08-10

**Authors:** Vladimir Matining, Mario Messina, Benedetta Sechi, Davide Moscatelli, Mattia Sponchioni

**Affiliations:** ^1^ Department of Chemistry Materials and Chemical Engineering “Giulio Natta” Politecnico Di Milano, Piazza Leonardo Da Vinci 32 Milan Italy

**Keywords:** 3D printing, adsorption equilibrium, chromatography, HETP, high‐throughput screening, microfluidics

## Abstract

3D printing is emerging as a promising fabrication technique for microfluidic devices. In this work, this technology was exploited in the development of a microfluidic chromatographic column with nominal volume of 54 µL. The microcolumn was packed with a cation exchange resin and characterized, using potassium iodide as a tracer, in terms of porosity (*ε* = 0.72), plate number, and asymmetry factor (0.8 < A_S_ < 1.8 for flowrates >50 µL/min). To showcase the potential of this microdevice, it was exploited in the characterization of the chromatographic behavior of lysozyme. The measured saturation capacity (*q* ^∞^= 88.14 g/L_resin_ at 340 cm/h) was in line with the manufacturer declaration (85–135 g/L at <500 cm/h). In addition, the effect of NaCl at different concentrations on the protein adsorption isotherm was characterized, demonstrating a Langmuir to anti‐Langmuir transition at concentrations ≥300 mM. The axial dispersion coefficient was finally determined ($D_{AX}$= 6.7 · 10^−9^ m^2^/s). In this way, the mcirofluidic column allowed to develop a comprehensive mechanistic model describing the transport of lysozyme in the chromatographic medium using only 30 µL of resin and <1 g of protein, addressing the issue of limited availability of biomolecules and streamlining the process development.

## Graphical Abstract and Lay Summary

Microfluidic tools are appealing solutions to speed up chromatographic process development. We exploited 3D printing to develop a microchromatographic set‐up, which was qualified in terms of porosity, HETP, and asymmetry factor. Using the 3D‐printed microcolumn, adsorption equilibrium, and mass transfer properties of lysozyme were characterized, leading to a detailed knowledge of its chromatographic behavior with minimal amount of material.

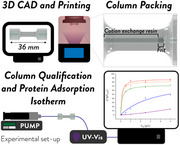

## 1 Introduction

In the 1980's, biopharmaceutical drugs emerged as revolutionary treatments for a wide range of diseases and started gaining interest in almost every branch of medicine. This is ascribed to their specificity and activity, marking a decisive improvement compared to conventional small‐molecule drugs [1, 2, 3]. To concretize these advantages, the biopharmaceutical companies are nowadays compelled to shorten the time‐to‐market and to reduce the costs of these biomolecules, with the aim of making them readily available to the patients. Intensification and efficient process development tools are essential in this direction, in particular for a field notoriously characterized by labor‐ and cost‐intensive experimentation [4, 5]. Significant advances have been achieved in the upstream processing (USP), where scale‐down models are currently extensively used to guide process development [6, 7, 8]. On the other hand, efficient and representative process development tools providing detailed knowledge about the chromatographic purification of biopharmaceuticals with limited time and material consumption are yet to be established [9]. This is becoming even more urgent with the progressive affirmation of personalized medicine, expected to bring to the market a handful of new molecules tailored to the patient's needs, with their own very specific physicochemical properties requiring detailed characterization [10, 11, 12, 13]. In this field, rapid and resource‐effective process development becomes even more crucial [14, 15]. Miniaturized systems have been proposed to cut the times and costs associated to the development of chromatographic operations. The golden standard is nowadays represented by robotic liquid handling stations (LHS), allowing testing multiple conditions in parallel and with reduced amount of material [16]. However, in the recent years, microfluidic devices emerged as attractive alternatives to LHS, due to the possibility of analyzing the system response in flow and in predominantly laminar conditions, which can be modeled with accuracy [17]. Polydimethylsiloxane (PDMS)‐based chips fabricated through soft lithography were demonstrated reliable scale‐down models for biochromatography, mainly due to their chemical inertness, low curing temperature, versatility, and low interaction with biological samples [18, 19, 20, 21]. As an example, in 2013 Chan et al. produced a microfluidic device comprising a packed column to separate dyes and biopolymers [22]. Another example refers to a chromatographic column developed by Ishida et al. to separate catechins [23]. Pinto et al. exploited this microfluidic system for the quantitative screening of different ligands in the separation of monoclonal antibodies, providing optimal operation windows [24]. Another recent example is a study by Javidanbardan et al. aimed at the experimental and numerical evaluation of the efficiency of a PDMS‐based microfluidic column, with a focus on its scalability [25]. Despite the great control over the geometrical features of the device, soft lithography comes with high fabrication complexity, long times for prototyping, the necessity of specialized operators, dedicated facilities, and expensive equipment. All these considerations make the fabrication of microfluidic chips expensive and difficult [18, 19, 21].

A bright solution is represented by 3D printing. This technique has gained traction in the recent years, due to its rapid prototyping, automation potential, incorporation of different materials in the same platform, and user friendliness [26, 27].

By leveraging these advantages, this study focused on the development of a 3D printed microchromatographic column to enable fast downstream processing development for biomolecules. After computer‐aided design (CAD) and 3D printing of the model, we packed the microcolumn with the strong cation exchange resin Eshmuno CPX. The packed bed was qualified in terms of porosity, height equivalent to a theoretical plate (HETP) and asymmetry factor at different flowrates using a small non‐binding tracer (i.e., potassium iodide). After having validated the efficiency of the microcolumn, its potential in the rapid and resource‐effective quantification of the adsorption equilibrium and mass transfer resistances was demonstrated for lysozyme, chosen as a model protein. We demonstrated that with minimal consumption of material and short time, a detailed understanding of its Langmuir adsorption isotherm, axial dispersion, and lumped rate constant describing the mass transfer in the stationary phase could be acquired. This information, in turn, allowed the establishment of a mechanistic model of the protein transport along the column, which could be used in the development of its chromatographic purification. This is essential for the model‐based design and control of efficient manufacturing processes, in line with the Quality‐by‐Design initiative.

## 2 Materials and Methods

### 2.1 Materials

Water washable photopolymerizable resin clear (Anycubic) was used as received. Ethanol (Sigma–Aldrich, >99.8%), 2‐propanol (IPA) (Sigma–Aldrich, >99.8%), potassium iodide (KI, Sigma–Aldrich), Eshmuno CPX (Millipore), poly(methyl methacrylate) beads with diameter 600 µm (PMMA, Thermo scientific), lysozyme from chicken egg white (Sigma–Aldrich), sodium hydroxide (NaOH, Sigma–Aldrich), sodium chloride (NaCl, Sigma–Aldrich), and sodium dihydrogen phosphate (NaH_2_PO_4_, Sigma–Aldrich) were of analytical‐grade purity and used as received unless specifically noted.

### 2.2 Computer‐Aided Design (CAD) and 3D Printing of the Column

**FIGURE 1:**
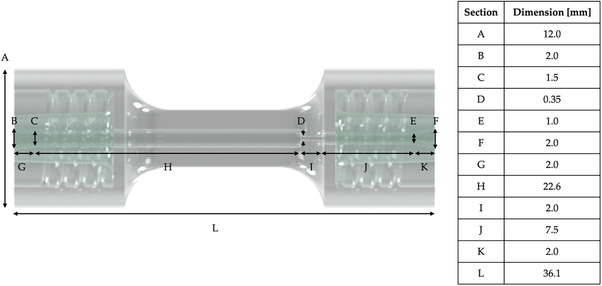
Schematic render of the microfluidic chromatography column with the corresponding geometrical features.

The microcolumn developed in this work (Figure 1) was designed using Autodesk Inventor 2023, and the resulting 3D model was sliced into layers using Chitubox that generates the print file. The inlet and outlet of the device were designed as luer‐locks to match common luer‐type fittings widely available commercially. In these sections, the inner diameter of the channel is 1.5 mm with a length of 9.0 mm. The inlet section is followed by the column compartment, with the same internal diameter and a total length of 22.6 mm. In order to retain the resin, the outlet section is obtained with a restricted channel having an internal diameter of 0.35 mm and a length of 2.0 mm.

The device was 3D printed using an easily accessible, commercial Anycubic Waterwash+ Clear Resin on an Elegoo Mars 2 Pro that uses UV photocuring technology with an integrated UV light source at 405 nm. The layer thickness was set at 0.05 mm, the bottom exposure time at 25 s, and normal exposure time at 1.5 s. After the printing process, the printed objects were immersed in an isopropanol bath and sonicated for 10 min and then further washed internally by injecting isopropanol manually through the channels.

### 2.3 Column Packing

The 3D printer used in the fabrication of the microfluidic column is associated to low commercial cost and user‐friendly technology, altogether leading to wide accessibility. However, this also results in a maximum resolution preventing to obtain microchannels with sufficiently small size to retain the cationic resin used, characterized by an average particle diameter of 50 µm. More advanced 3D printers could solve this criticality, but at the expense of higher machine costs, and possibly more complex procedures. Hence, in our case, it was necessary to use a frit system allowing to retain the cationic resin. Therefore, the column packing was performed in two steps. First, PMMA particles with a diameter of 600 µm were introduced in the column using a glass pipette to fill approximately a length corresponding to the diameter of the column. This PMMA layer is used as frit to retain the resin beads. Afterward, the resin slurry Eshmuno CPX, diluted to 1:5 with ethanol to ease the introduction in the column, was packed by using a positive pressure through a syringe pump injecting water at 0.5 mL/min for 45 min. The column packing was visually tracked on a WILD Stereomicroscope Model M8 with a DFC 290 camera and a magnification of 20×.

### 2.4 Column Characterization

The column qualification was performed by connecting the microfluidic device to a Harvard Apparatus 33 dual infuse/withdraw syringe pump at the inlet and to an inline UV detector (AZURA Detector MWD 2.1L, Knauer) with a microflow cell (Semi‐preparative 3 mm UV Flow Cell) with internal volume of 2 µL and equipped with fiber optic probes at the outlet, in order to monitor the UV absorbance of the eluate during time. The UV signal was measured and recorded using ClarityChrom Workstation (Knauer).

Breakthrough experiments with an inert tracer (KI) were performed to determine the total void fraction of the column. First, the dead volume (*V*_0_) given by the capillaries and the flow cell was measured by dispensing a 0.1 M KI solution at 0.1 mL/min to the setup with a zero‐dead volume connector replacing the microcolumn until a plateau in the UV signal, measured at 265 nm, was recorded. The same experiment was then repeated by connecting the empty column to the system to determine the empty column volume (*V_C_*). Finally, the packed column was plugged in for assessing the bed porosity (*ε*). Equation (1) shows the mass balance applied to the system for the quantification of its characteristic volumes.

(1) $$QC_{in}\tau =Q\int_{0}^{\tau }C\left(t\right)\;dt+\left[V_{0}+\varepsilon V_{C}\right]C_{in}$$

where *Q* is the volumetric flowrate, *C_in_* the concentration of KI fed to the system, *τ* the loading time and *C*(*t*) the instantaneous outlet concentration. In the experiment without column (*V_C_* = 0), the equation was solved for *V*_0_. When connecting the empty column (*ε* = 1), through this equation it was possible to measure *V_C_*. Finally, with an additional breakthrough experiment on the packed column, the equation was solved for *ε*.

Pulse injections were then performed to calculate the HETP and number of plates (N) of the column. These experiments were performed by injecting a constant volume (15 µL) of a tracer solution (0.1 M of KI) into the column at different flowrates (0.05, 0.1, 0.15, and 0.2 mL/min) for the mobile phase (water). The KI concentration during time in the effluent from the column was measured via UV spectroscopy at 265 nm. To separate the contribution from the dead volume and from the column on the measured peaks, the data were analyzed by modeling the system as a series of capillaries and microcolumn, assuming negligible radial gradients and non‐binding conditions, which led to Equation (2).

(2) $$\;\frac{\partial C\left(t,z\right)}{\partial t}=-v\frac{\partial C\left(t,z\right)}{\partial z}+D_{ax}\frac{\partial^{2}C\left(t,z\right)}{\partial z^{2}}\;$$

where *v* is the interstitial velocity, *D_ax_* is the axial dispersion coefficient and *z* is the longitudinal coordinate of the system. The equation was integrated assuming an initial concentration equal to 0 all along the system and Danckwerts boundary conditions, as reported in Equations (3)–(5).

(3) $$C\;\left(t=0,z\right)=0$$

(4) $$vC\left(t,z=0\right)-D_{ax}\;{\left.\frac{dC}{dz}\right|}_{z=0}=vC_{in}$$

(5) $$\;{\left.\frac{dC}{dz}\right|}_{z=L}=0$$

The axial dispersion coefficient (*D_ax_*) for the capillaries was preliminarily determined by fitting the breakthrough experiment with the zero‐dead volume connector replacing the column. The genetic algorithm routine in MATLAB was exploited to minimize the root‐mean square error between the simulated and the experimental curve and run with a lower boundary of 1 · 10^−8^ m^2^/s and upper boundary of 1 · 10^−4^ m^2^/s. After that, the axial dispersion in the column was regressed in the same way. Based on these estimated parameters, pulse injections at different flow rates were simulated and the agreement with the experimental results was verified by comparison with the peaks measured at the tested flow rates. After having validated the model results, this was used to calculate the HETP at different interstitial velocities according to Equation (6).

(6) $$HETP=L\frac{\sigma^{2}}{\mu_{1}^{2}}\;$$

where L is the length of the column, and σ^2^ the variance of the peak calculated according to Equation (7).

(7) $$\;\sigma^{2}=\frac{\int_{0}^{\infty }C*{\left(t-\mu_{1}\right)}^{2}\;dt}{\int_{0}^{\infty }C\;dt}$$

Finally, μ_1_ is the first order moment, expressed as in Equation (8).

(8) $$\;\mu_{1}=\frac{\int_{0}^{\infty }C*t\;dt}{\int_{0}^{\infty }C\;dt}\;$$

The number of theoretical plates was then estimated according to Equation (9).

(9) $$N=\frac{L}{HETP}\;$$

The Van Deemter plot was then constructed from the HETP, and N calculated from the experimental data. The plot was extended to a wider range of flowrates from 0.01 to 0.21 mL/min by simulations through the calibrated model.

From the same pulse experiments, the peak asymmetry factor (A_S_) was calculated according to Equation (10).

(10) $$\;A_{S}=\frac{t_{2}-\mu_{1}}{\mu_{1}-t_{1}}\;$$

where *t*_1_ is the time at which the signal reaches 10% of the peak maximum in the front, while *t*_2_ is the time at which the signal decreases to 10% of the peak maximum in the tail.

### 2.5 Quantification of Lysozyme Adsorption Equilibrium and Mass Transfer Parameters

The isotherm parameters of lysozyme, a model protein widely used in the biopharmaceutical industry, were determined through breakthrough adsorption tests using the packed microcolumn.

The mobile phase was a 20 mM solution of NaH_2_PO_4_, whose pH was regulated to 6.1 with 5 M NaOH. The strip buffer used to clean the column was a 25 mM NaH_2_PO_4_ + 1 M NaCl solution at pH 6.1.

Initially, the bed porosity of the column for lysozyme was determined from Equation (1) by conducting breakthrough experiments at 1 g/L in strip buffer to ensure non‐binding conditions, and at a flowrate of 0.1 mL/min.

Then, to measure the adsorption isotherm, lysozyme solutions at different concentrations in the mobile phase (0.05, 0.1, 0.5, 1, and 5 g/L) were prepared and dispensed to the system at 0.1 mL/min until a plateau in the UV signal measured at 274 nm was observed. After each test, 10 column volumes (CV) of the strip solution were dispensed into the column to desorb the protein, followed by re‐equilibration with the mobile phase for 10 CV. To determine the average concentration of lysozyme in the solid phase (q*) in equilibrium with the liquid phase, the mass balance reported in Equation (11) was applied.

(11) $$C_{in}Q\tau =Q\int_{0}^{\tau }Cdt+C_{in}\left(V_{0}+\varepsilon V_{c}\right)+q^{*}\left(1-\varepsilon \right)V_{c\;}$$

Based on the measured q* at the different inlet lysozyme concentrations, the parameters of the Langmuir isotherm (Equation 12), namely, the adsorption equilibrium constant (K) and resin saturation capacity (*q* ^∞^) were quantified by linear regression.

(12) $$\;q^{*}=q^{\infty }\;\frac{KC_{eq}}{1+KC_{eq}}\;$$

This study was extended to different modifier (NaCl) concentrations in the mobile phase, as it strongly affects the adsorption equilibrium. In general, when applied to the description of protein adsorption, the parameters *q* ^∞^ and *K* depend on the composition of the mobile phase (i.e., salt concentration, presence of organic modifiers, pH, etc.) [28]. In the case of protein adsorption on ion exchangers, the maximum adsorption capacity and the equilibrium constant are a function of the salt concentration in the mobile phase, while the functional form of the isotherm remains unchanged [29]. To find the optimal separation conditions, the accurate modelling of the protein retention behavior based on the mobile phase composition is important in chromatography [30].

To describe the changes in the retention time of lysozyme with the concentration of NaCl present in the mobile phase and acting as modifier, the linear solvent strength (LSS) model was used. Developed by Snyder and Dolan in the 1990s, the LSS introduces an exponential dependence of *q* ^∞^ and *K* on the NaCl concentration (φ(mol/L)) [29, 30, 31], as in Equations (13)–(14).

(13) $$K=K_{0}\;\cdot exp\left(-S_{K}\varphi \right)$$

(14) $$\;q^{\infty }=q_{0}^{\infty }\;\cdot exp\left(-S_{q}\varphi \right)$$

where *K*_0_ (L/g) and $q_{0}^{\infty }$ (g/L_resin_) are the adsorption equilibrium constant and saturation capacity at φ  =  0, respectively, while *S_K_* (L/mol) and *S_q_* (L/mol) are the two parameters expressing the sensitivity of *K*_0_ and $q_{0}^{\infty }$ to the modifier concentration [32].

To account for the effect of NaCl with the LSS model, Equation (12) can therefore be modified providing the generalized form of the Langmuir isotherm (Equation 15).

(15) $$\;q^{*}=\frac{a_{0}\cdot exp\left(-S_{a}\varphi \right)\cdot C_{in}}{1+K_{0}\cdot exp\left(-S_{K}\varphi \right)\cdot C_{in}}\;$$

where $a_{0}=q_{0}^{\infty }\;\times K_{0}$ and *S_a_* = *S_q_*  + *S_K_*.

To experimentally evaluate the parameters *a*_0_, *K*_0_, *S_a_*, and *S_K_* for lysozyme, a series of breakthrough studies at different NaCl concentrations in the mobile phase were performed. Four different mobile phases were investigated, containing 50, 100, 300, and 500 mM NaCl. Each mobile phase was used to prepare lysozyme solutions with concentrations of 0.50, 1.00, 2.50, and 5.00 g/L. Breakthrough experiments were performed in each condition. Three breakthrough profiles were measured for each mobile phase composition and lysozyme concentration. The packed microfluidic column was at first equilibrated with 10 CV of the investigated mobile phase. The lysozyme solution was then pumped inside the column until breakthrough was achieved. Lastly, before proceeding with a new run, a 10 CV strip step was performed to clean the resin.

By integrating the breakthrough profiles obtained and applying the mass balance on the system reported in Equation (11), the average concentration of lysozyme adsorbed on the stationary phase at equilibrium (q*) was quantified for any given mobile phase composition and feed concentration tested.

The results were first fit to the Langmuir isotherm model to obtain the parameters *q* ^∞^ and *K* for each of the four mobile phases tested. A linear regression on Equations (13) and (14) was then applied to determine the isotherm parameters *a*_0_, *K*_0_, *S_a_*, and *S_K_* for lysozyme.

Finally, the axial dispersion coefficient (D_ax_) and the lumped mass transfer rate constant (K_LDF_) were determined by regression of the experimental breakthrough curves. In particular, the equilibrium‐dispersive model obtained by neglecting radial concentration gradients and describing the mass transport into the stationary phase through the linear driving force model was considered, as reported in Equation (16).

(16) $$\begin{array}{cc} \frac{\partial C\left(t,z\right)}{\partial t} & =-v\frac{\partial C\left(t,z\right)}{\partial z}+D_{ax}\frac{\partial^{2}C\left(t,z\right)}{\partial z^{2}}\\ & \;-\frac{1-\varepsilon }{\varepsilon }K_{LDF}\left(q^{*}-q\left(t,z\right)\right) \end{array}$$

where q(t,z) is the local average concentration of lysozyme in the solid phase.

The equation was integrated using the same initial and boundary conditions as reported in Equations (3)–(5), with the addition of an initial condition considering concentration in the solid phase equal to 0 all along the column.

The data fitting was performed using the genetic algorithm routine in MATLAB, minimizing the root‐mean square error between the simulated and the experimental breakthrough curves. For D_ax_ a lower boundary of 1 · 10^−10^ m^2^/s and an upper boundary of  1· 10^−8^ m^2^/s were considered. For K_LDF_, we considered the linear proportionality with the lysozyme feed concentration (Equation 17) proposed by Silva et al. [33].

(17) $$\;K_{LDF}=\delta \cdot C_{in}\;$$

where 𝛿 is a constant that was regressed by imposing a lower boundary of 1 · 10^−4^ L/ (g s) and an upper boundary of 1 · 10^−2^ L/ (g s).

## 3 Results and Discussion

### 3.1 3D Printed Chromatographic Microcolumn and Resin Packing

**FIGURE 2:**
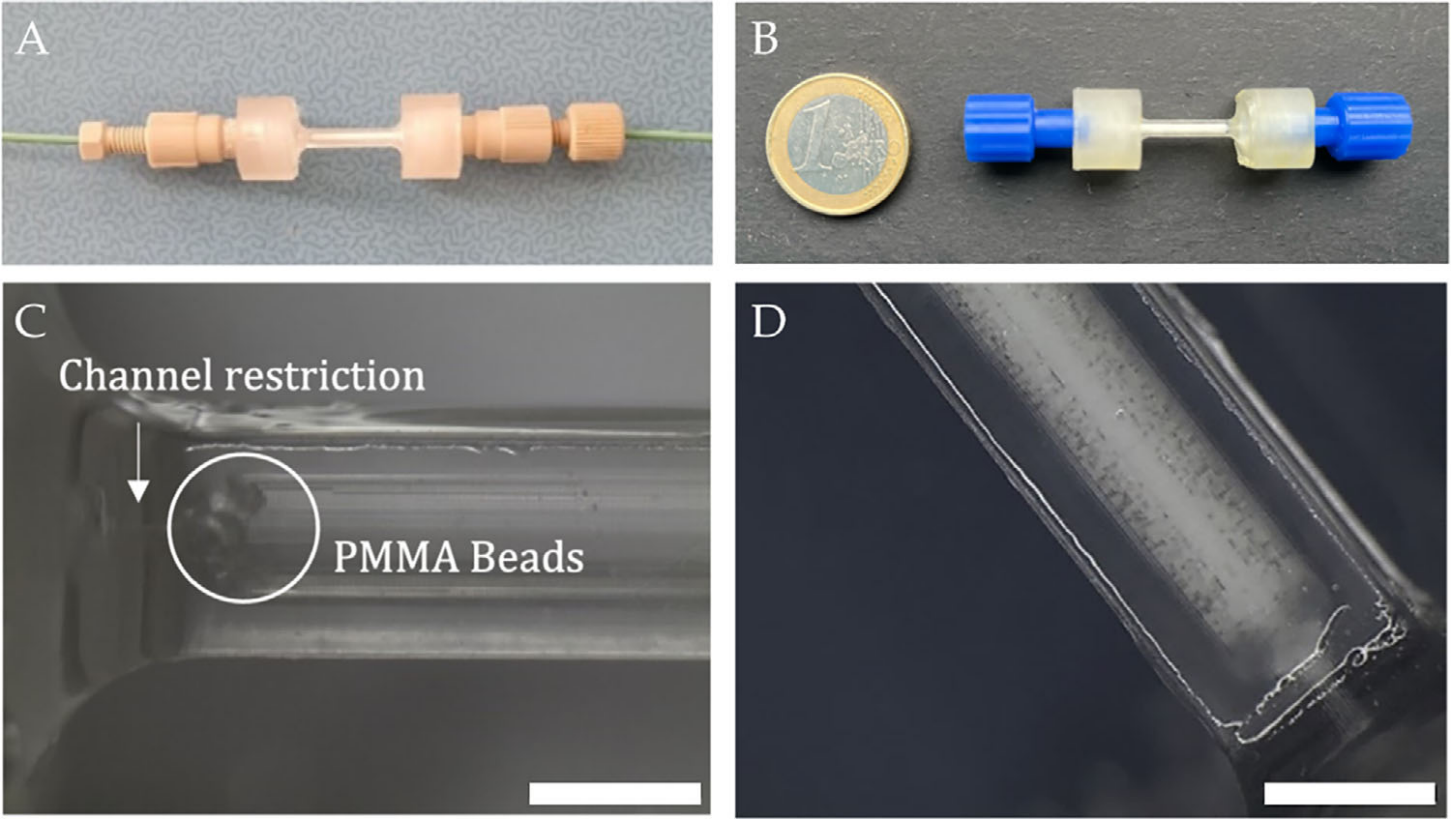
(A) Connection of the 3D printed column to capillaries using PEEK female luers and fittings. (B) Dimensional comparison of the packed 3D printed column with a coin. (C) Stereomicroscope pictures of the column during packing with PMMA beads (magnification 20×, scale bar = 2 mm) and (D) with the cationic resin Eshmuno CPX (magnification 20×, scale bar = 2 mm).

The microfluidic chromatographic column was 3D printed on a digital light photopolymerization printer according to the CAD reported in Figure 1. The design of the column allowed for the use of generic female luer fittings to connect capillaries as shown in Figure 2A. The small column size, with nominal bed volume of 54 µL can be appreciated through a comparison with a 1 Euro coin in Figure 2B. This set up allowed to minimize the leakages that are caused by the high pressure usually generated within the system. The mechanical properties of the cured resin used in the fabrication of the microfluidic column are reported in Table 1 and compared to FormLabs Durable. The latter was implemented by Roca et al. in the fabrication of a microfluidic device packed with C18 resin beads [34]. In their work, the failure of the device was observed at 1.1 mL/min, with corresponding backpressure of 40 bar, after prolonged usage. Herein, the resin used shares similar mechanical properties but the flowrates tested were at least five times lower than the cited breaking point value, which ensured the durability of the device.

**TABLE 1:** Mechanical properties of the 3D printing resins used in this work and in [34] as declared by the manufacturer.

Parameter	Anycubic Waterwash Resin +	FormLabs Durable
**Tensile strength [MPa]**	30–45	31.8
**Flexural modulus [GPa]**	1.50–1.60	0.82
**Notched impact strength [J/m]**	>50–60	109

This microcolumn was first packed with a thin layer of PMMA beads, retained by the restricted outlet as shown in Figure 2C. PMMA was chosen because of its inertness with respect to biological material, as already demonstrated by other authors [35]. This frit layer, in turn, was essential to retain the smaller resin particles, which were used to pack the remaining volume of the column (Figure 2D). Indeed, with the 3D printer used, it was not possible to obtain channels with inner diameter small enough to retain 50 µm beads. This high resolution could be potentially reached with more sophisticated devices, at the expense of higher costs, which could mine the accessibility of this technology.

**FIGURE 3:**
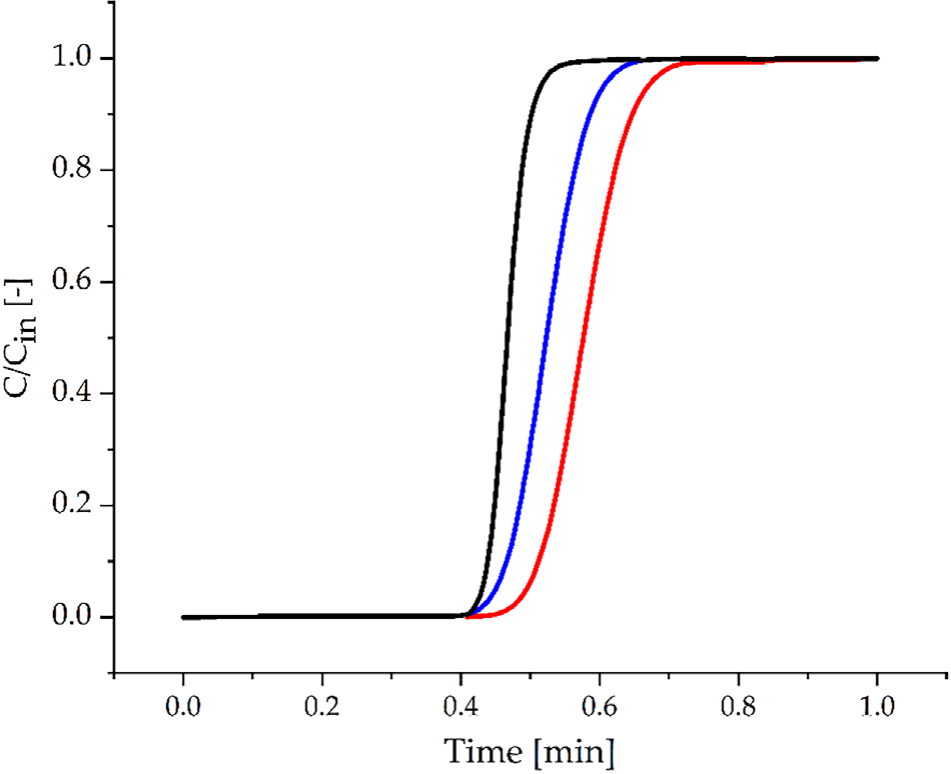
Experimental breakthrough curves performed on the dead volume of the system by replacing the microcolumn with a zero‐dead volume connector (▬), on the empty column (▬), and on the packed column (▬).

The packed bed was characterized in terms of column volume (*V_C_*) and bed porosity (*ε*) through breakthrough experiments using KI as inert tracer. The breakthrough curves obtained for the three cases of: (i) zero‐dead volume replacing the column, (ii) empty column, and (iii) packed column are shown in Figure 3.

The steepness of the breakthrough curve associated to the dead volume is the highest, as expected by the limited non‐ideality in this segment compared to that observed in the microcolumn. In fact, the axial dispersion in the interstices between the resin particles as well as the diffusion into the bead pores broaden the band, making the breakthrough curve shallower. From these curves and exploiting Equation (1), the characteristic volumes of the system can be measured. These are summarized in Table 2.

**TABLE 2:** Theoretical and calculated volumes for the 3D printed microcolumn.

	Expected volume (CAD)	Dead volume (*V*_0_)	Empty column (*V_C_*)	Packed column (*V*_packed_)
**Volume [µL]**	54	413.0	75.5	54.4

It can be observed that the measured empty column volume (V_C_) is larger than the expected value from the CAD project. This may reflect some inaccuracy in the 3D printing at such small level of detail. Indeed, less than 0.2 mm change in the actual column diameter is sufficient to justify the observed difference in volume. From the breakthrough curve on the packed column, the volume of the empty fraction of the column was calculated, which provided a way for estimating the column porosity, *ε* = 0.72. For comparison, in a work by Cingolani et al. [36], conventional GE Tricorn columns with 50 mm length and 5 mm diameter were packed with the same Eshmuno CPX resin. The columns were characterized using dextran tracers with minimum molecular weight of 1 kDa. In that case, the porosity was 0.70, very similar to the value measured in this work with our microcolumn at a fraction of the time. It is worth highlighting that the dead volume is more than five times larger than the column volume. This implies that, while for traditional chromatographic equipment the influence of V_0_ on Eddy dispersion and other phenomena leading to band broadening can be reasonably neglected with respect to the contribution from the packed column, this is no longer the case when dealing with microfluidic devices. The effect of the dead volume on retention time and peak shape is more pronounced, as presented in the following section. Therefore, this needs to be properly accounted for when aiming at an accurate measurement of the column performances.

### 3.2 Column Efficiency

The efficiency of the 3D printed microcolumn was investigated through a series of pulse injections performed at different flowrates, with the aim of quantifying the HETP and peak symmetry. To decouple the effect of the dead volume and of the microfluidic column, experimental breakthrough curves with and without the column connected were regressed with the dispersive model (Equation 2) simulating the two systems in series. Through this approach, the axial dispersion coefficient in the dead volume (D_ax,V0_ = 3.2 · 10^−6^ m^2^/s) and in the column (D_ax,C_ = 8.01 · 10^−6^ m^2^/s) could be estimated. From these values, the Peclet number (Pe) was evaluated to characterize the relative importance of convective and diffusive transport [37]. The definition is provided in Equation (18).

(18) $$Pe=\frac{L_{C}\cdot v}{D_{AX}}\;$$

where *L_C_* (m) is the characteristic length of the system, *v* (m/s) is the interstitial velocity, and $D_{AX}$ (m^2^/s) is the axial dispersion coefficient. In the context of this work, by considering as characteristic length the one of the microfluidic column, a Pe of 2.7, a value greater than 1 is obtained. This indicates the dominance of convective transport within the column.

**FIGURE 4:**
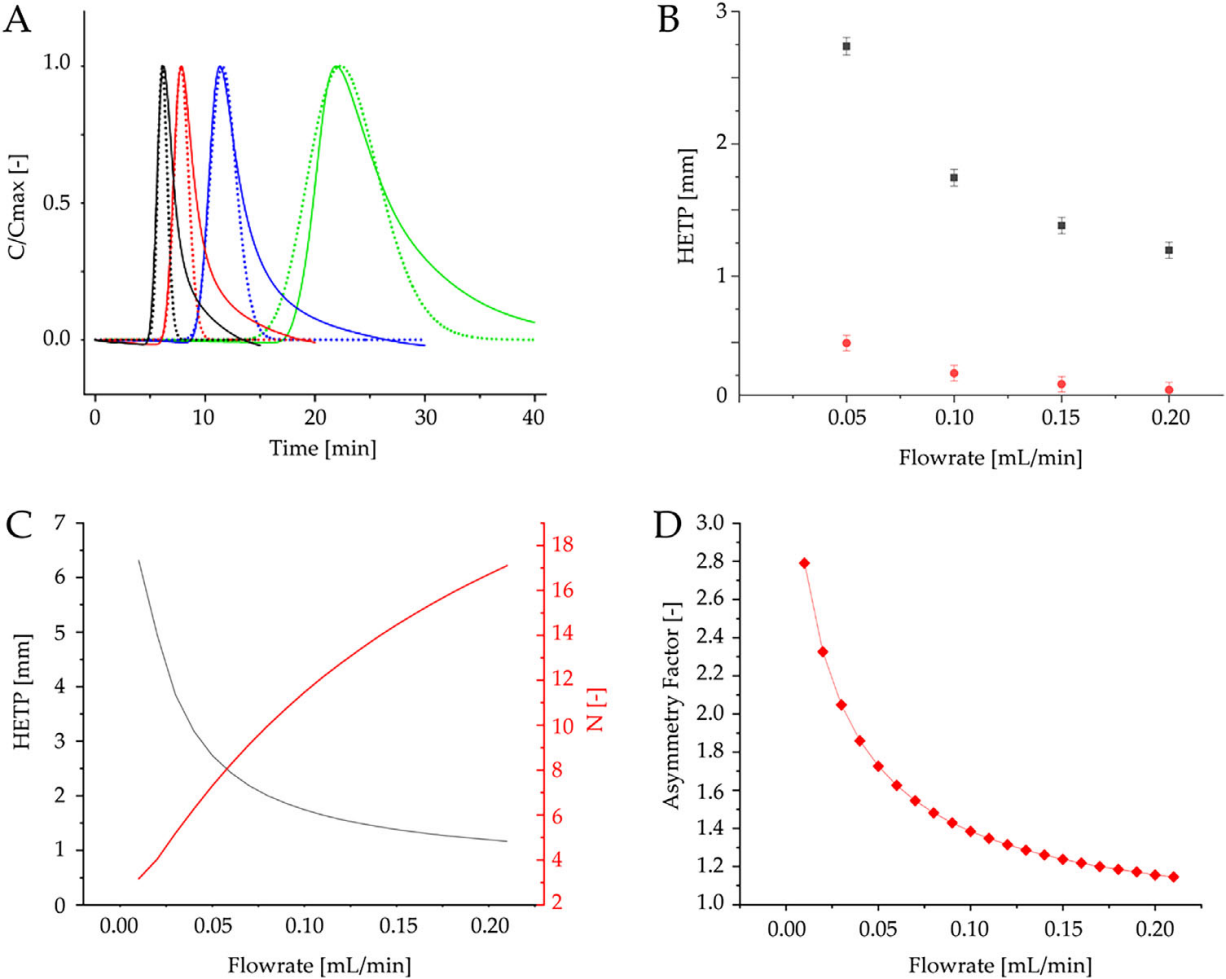
(A) Pulse injections performed at different flowrates on the 3D printed microfluidic column with the inert tracer KI. The solid lines refer to the experimental results, while the dotted lines refer to the model predictions. ▬ 0.05 mL/min, ▬ 0.10 mL/min, ▬ 0.15 mL/min, ▬ 0.20 mL/min; (B) HETP as a function of the flowrate for the entire setup, comprising the column and dead volume (black squares), and only for the microcolumn (red circles); (C) HETP and number of theoretical plates as a function of the flowrate referred to the 3D printed microcolumn from pulse injections; (D) Asymmetry factor as a function of the flowrate for the 3D printed microcolumn.

The reliability of the estimated axial dispersion coefficients was then verified by simulating experimental pulse injections at 0.05, 0.10, 0.15, and 0.20 mL/min, as shown in Figure 4A.

It can be noticed that the model reproduces the experimental mean residence time and peak front with high accuracy. On the other hand, a pronounced tail was observed in the experimental pulses, which was attributed to an imperfect pulse regulation by the selection valve. Given the good agreement between the experimental and model results, the latter was exploited to assess the contribution of the microcolumn to the total observed HETP, as shown in Figure 4B. It is noticeable that the HETP for the column alone (red dots) is significantly smaller than that for the entire system (black squares), highlighting once more the relevant contribution of the dead volume to the data obtained when using a microfluidic device. In the work by Cingolani et al. [36] increasing HETP values ranging from 1 to 2 mm were calculated when analyzing 12 kDa dextran tracers with flowrates from 0.5 and 2 mL/min. Despite these flowrates are above the range considered in this study, the absolute values of HETP were still comparable, thus supporting the reliability of this microcolumn in the estimation of the column efficiency.

In addition, the model was used to simulate pulse injections in a broader range of flowrates and define the Van Deemter plot in terms of HETP and number of plates N as a function of the flowrate, as reported in Figure 4C.

In this plot, the traditional contributions from axial diffusion and hydrodynamic dispersion at low and medium flowrates, respectively, can be appreciated. On the other hand, intraparticle mass transfer limitations were not observed, as expected from the use of a small ion as a tracer, which quickly diffuses inside the particle pores.

Finally, Figure 4D shows the asymmetry factor as a function of the flowrates. It can be noted that higher flowrates result into more and more symmetric peaks, with A_S_ falling in the range typically considered as acceptable (0.8 < A_S_ < 1.8) for flowrates >50 µL/min.

### 3.3 Characterization of Lysozyme Adsorption

To showcase the application of this 3D printed microcolumn, we investigated the adsorption equilibrium of lysozyme, a well‐known model protein, on the cation exchanger Eshmuno CPX. First, the total porosity for lysozyme was measured through a breakthrough experiment in non‐binding conditions (i.e., 25 mM NaH_2_PO_4_ + 1 M NaCl). This was *ε* = **0.71**. This value nicely agrees with the porosity measured by Cingolani et al. in a 1 mL prepacked column using 12 kDa dextran, which was found between 0.6 and 0.7 [36]. Furthermore, in a study by Steinebach et al., the porosity of 12 different cation exchange resins packed in a 5 × 50 mm column were determined via breakthrough tests using lysozyme (5 g/L) at flowrates 0.5 and 2 mL/min. In all the situations, the porosity was in the range 0.63–0.9 [38]. Since the porosities measured with a small tracer like KI and lysozyme were very similar, we concluded that the latter can access most of the pores in this resin.

**FIGURE 5:**
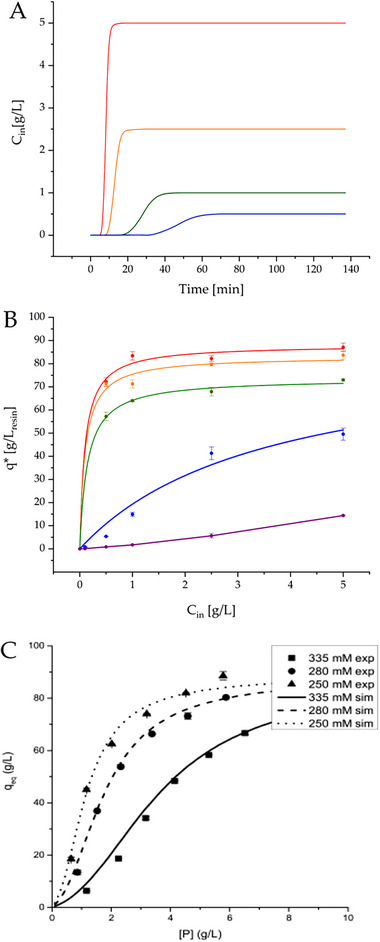
(A) Breakthrough curves of lysozyme over the packed column at different lysozyme concentrations: ▬ 5.0 g/L, ▬ 2.5 g/L, ▬ 1.0 g/L, ▬ 0.5 g/L. (B) Lysozyme adsorption isotherms at varying mobile phase modifier concentrations: ▬ 0 mM NaCl, ▬ 50 mM NaCl, ▬ 100 mM NaCl, ▬ 300 mM NaCl, ▬ 500 mM NaCl. Symbol = Experimental results; Lines = Fitting to a Langmuir isotherm. (C) Adsorption isotherm of lysozyme on conventionally packed Eshmuno CPX in 5 mm × 50 mm column with different NaCl modifier concentrations (335 mM, 280 mM, 250 mM). Figure reproduced from Khalaf et al. [39] with permission from Elsevier.

Then, the average protein concentration in the stationary phase in equilibrium with the liquid was measured through a series of breakthrough tests at different feed concentrations in a 20 mM NaH_2_PO_4_ buffer. Figure 5A shows the breakthrough curves for the four concentrations considered (0.50–5.00 g/L). Through the integration of these breakthrough curves, and using Equation (11), the concentration of the lysozyme adsorbed on the stationary phase at equilibrium (*q* ^*^) was determined as reported in Table 3, as well as in Figure 5B (red line). Here, a typical Langmuirian behavior can be appreciated. Therefore, the Langmuir isotherm (Equation 12) was linearized to regress the monolayer saturation capacity and equilibrium constant.

**TABLE 3:** Lysozyme concentration in the stationary phase in equilibrium with the liquid for each inlet concentration injected. The values are reported as average ± standard deviation of three replicates.

C_in_[g/L]	***q*** ^*^ [g/L_resin_]
0.50	72.16 ± 1.83
1.00	83.41 ± 1.83
2.50	82.19 ± 1.23
5.00	87.07 ± 1.83

These were *q* ^∞^ = 88.14 g/L_resin_ and *K* = 10.05 L/g, respectively. In the same work by Steinebach et al. [38], the saturation capacities measured for lysozyme using 12 different cation exchange resins in traditional prepacked columns were in the range 55–240 g/L, which is in good agreement with the value determined with the microfluidic column used herein. Furthermore, it can be noted that the resin saturation capacity calculated at a linear velocity of 340 cm/h is in line with the dynamic binding capacity declared for Eshmuno CPX by the manufacturer (85–135 g/L for flowrates up to 500 cm/h), as well as with that reported by Kim et al. for lysozyme in a MiniChrom Column Eshmuno CPX (8 × 100 mm), namely, 109 g/L [40]. This further confirms the reliability of this microfluidic device in the characterization of adsorption equilibria using only minimal amounts of resin and protein. The high value of equilibrium constant and the slope of the linear portion of the isotherm indicates favorable adsorption behavior of lysozyme to the resin.

A similar investigation was extended to different NaCl concentrations in the buffer, to clarify its role on the adsorption equilibrium for lysozyme. Breakthrough tests at varying lysozyme feed concentration allowed for the determination of the adsorption isotherm at each NaCl concentration tested. These are shown in Figure 5B. From their regression, the saturation capacity and equilibrium constant were obtained as summarized in Table 4.

**TABLE 4:** Langmuir isotherm parameters for lysozyme at different NaCl modifier concentrations.

NaCl concentration [mM]	*q* ^∞^ [g/L_resin_]	*K* [L/g]
0	88.14	10.04
50	83.48	8.99
100	73.59	6.80
300	87.82	0.28
500	−23.91	−0.08

From Figure 5B and the parameters estimated in Table 4, it is possible to observe that lysozyme follows a typical Langmuir behavior up to 100 mM NaCl in the mobile phase. For higher modifier concentration, a different adsorption behavior can be observed. In the 300 mM NaCl solution, a quasi‐sigmoidal adsorption isotherm can be deduced from the experimental data, with an inflection point at approximately 0.5 g/L. In the case of 500 mM NaCl, a complete change in isotherm type is observed as a clear anti‐Langmuir behavior can be recognized, with an almost linear trend up to 1 g/L, followed by a concave‐up increase at higher concentrations. This behavior is evident from the negative values of saturation capacity and equilibrium constant regressed in this case, which of course bear no physical meaning but underline the deviation from the Langmuir model.

From a physical point of view, the sigmoidal transition observed in the isotherm at 300 mM NaCl as the lysozyme concentration in the mobile phase increased may be an indication of a multilayer adsorption taking place on the resin. Khalaf et al. observed and reported this behavior for lysozyme on polyelectrolyte brushes (PEB) resins, developing a complex adsorption isotherm to account for the possible formation of multiple layers during adsorption [39]. The authors concluded that the protein is initially adsorbed at a free site close to the interface between the PEB and the free pore volume of the stationary phase. As a second protein approaches, for its adsorption to take place, the first protein is pushed further inside the PEB phase. As several proteins approach the PEB, this process can occur several times. However, considering steric hindrance and the hydrodynamic radius of lysozyme, a maximum of three layers can be formed. Furthermore, the three isotherms with Langmuir behavior observed in this work are in line with the observation by Khalaf et al. in which, as the modifier concentration increases, the steepness of the linear portion of the isotherm decreases as well as the absolute values, as shown in Figure 5C. This unexpected literature validation confirms the reliability of this 3D printed microcolumn as scale‐down model of a chromatographic separation, providing important insights into the adsorption behavior of a biomolecule with very limited amount of material consumed.

Considering the adsorption isotherm determined at 500 mM NaCl, the occurrence of an anti‐Langmuir behavior can be associated to strong protein–protein interactions leading to a multilayer adsorption [41]. From a physical standpoint, in a study by Chen et al., it was concluded that the presence of salts increases the protein binding in hydrophobic dominated processes, thus reducing binding affinity as the concentration of the salt modifier increases in a multi‐interaction system [42]. Furthermore, the Langmuir model utilized in this study typically accounts for adsorption isotherms at constant pH and ionic strengths. Indeed, it has been stated that even modified Langmuir models could not fully describe the influence of ionic strength in the protein adsorption behaviors. This could be due to the assumed adsorption reaction between proteins and the adsorption site in the Langmuir model, which unproperly considers the multi‐pointed nature of protein binding, and the steric hindrance caused by pre‐adsorbed solutes such as in the case of salts [42].

### 3.4 Evaluation of the Mass Transfer Parameters

**FIGURE 6:**
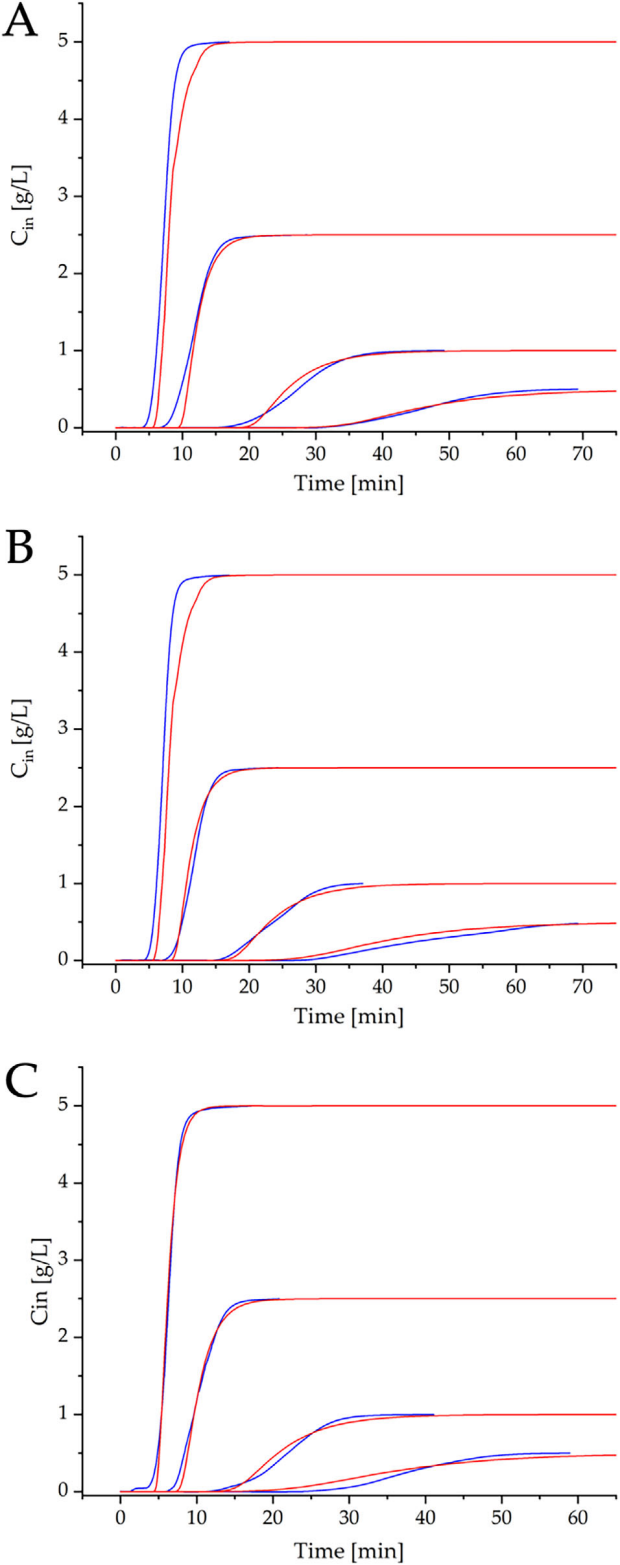
Experimental breakthrough curves (▬) and model predictions (▬) at different feed concentrations (0.50, 1.00, 2.50, and 5.00 g/L) and modifier concentrations: (A) 0 mM NaCl, (B) 50 mM NaCl, (C) 100 mM NaCl.

The equilibrium‐dispersive model was adopted to describe the transport of lysozyme in the column, whilst its accumulation in the solid phase is accounted for through the linear driving force approximation. While the equilibrium concentration in the stationary phase was evaluated in detail through the adsorption studies reported in the previous section, the integration of Equation (16) required the axial dispersion coefficient and the mass transfer parameter *K_LDF_*. To calibrate the model, the breakthrough curves for lysozyme measured at different feed concentration and modifier content were regressed using a genetic algorithm (Figure 6).

Before conducting these calculations, the contribution of the dead volume of the system to the axial dispersion was accounted for through breakthrough experiments in non‐binding conditions. From data fitting, the axial dispersion coefficient for the column resulted $D_{\mathrm{AX}}$= 6.7 · 10^−9^ m^2^/s (Pe = 3.2 · 10^3^). In a similar research conducted by Morgenstern et al., the authors found $D_{\mathrm{AX}}$= 6.7 · 10^−8^ m^2^/s (Pe = 77) using a pre‐packed Toyoscreen column (6.4 mm × 30 mm) with TOYOPEARL GigaCap S‐650 M cation exchange resin [43]. This good agreement was again considered a proof of the reliability of this microcolumn.

On the other side, we adopted for *K_LDF_* the correlation with the inlet lysozyme concentration reported in Equation (17) [33]. By looking at Figure 6, with a single value of 𝛿 = 3 · 10^−3^ L/(g s) we could reproduce the breakthrough curves at different salt and lysozyme concentrations with good accuracy.

Using this 3D printed column, it is then possible to rapidly develop a mechanistic model to describe the transport and retention of a protein in a chromatographic column, thus streamlining the process development stage.

## 4 Conclusions

In this study, the possibility of rapidly fabricating a microfluidic chromatographic column was explored using 3D printing. This technique may lead to important improvements in the establishment of reliable scale‐down models for chromatographic operations, being cheap and accessible to non‐specialized personnel. The potential of the 3D printed microcolumn in shedding light on the fundamental principles of protein adsorption was demonstrated considering lysozyme as a model biomolecule. With minimum amount of material consumed and limited experimental effort, the detailed description of the Langmuir isotherm, including the impact of NaCl concentration on the equilibrium constant and saturation capacity, could be accessed. Indeed, in the fabrication of one column, around 30 µL of cation exchange resin was used, compared to a typical 5 mL prepacked column, leading to more than 100 times material reduction. More importantly, the amount of sample used was also minimized. Indeed, considering the time needed to reach the plateau value for each of the breakthrough curves shown in Figure 5A, less than 10 mg of lysozyme was utilized. This suggests the opportunity of extending the application of these devices in the characterization of other biomolecules, typically available in small amounts, with the aim of shortening the process development time notoriously associated to chromatographic operations.

## Author Contributions

**V.M**.: Investigation, Data Curation, Writing – Original Draft; **M.M**.: Data Curation, Software; **B.S**.: Data Curation, Validation, Software; **D.M**.: Supervision; **M.S**.: Conceptualization, Supervision, Funding Acquisition, Writing – Review and Editing.

## Conflicts of Interest

The authors declare no conflict of interests.

## Data Availability

The data that support the findings of this study are available from the corresponding author upon reasonable request.
